# Proliferative Signals in Chronic Lymphocytic Leukemia; What Are We Missing?

**DOI:** 10.3389/fonc.2020.592205

**Published:** 2020-10-08

**Authors:** Marco V. Haselager, Arnon P. Kater, Eric Eldering

**Affiliations:** ^1^ Department of Experimental Immunology, Academic University Medical Center, location Academic Medical Center, University of Amsterdam, Amsterdam, Netherlands; ^2^ Lymphoma and Myeloma Center Amsterdam, LYMMCARE, Amsterdam, Netherlands; ^3^ Cancer Center Amsterdam, LYMMCARE, Amsterdam, Netherlands; ^4^ Amsterdam Infection & Immunity Institute, Amsterdam, Netherlands; ^5^ Department of Hematology, Academic University Medical Center, location Academic Medical Center, University of Amsterdam, Amsterdam, Netherlands

**Keywords:** chronic lymphocytic leukemia, proliferation, micro-environment, CD40, toll-like receptor, crosstalk

## Abstract

Chronic lymphocytic leukemia (CLL) cells cycle between lymphoid tissue sites where they actively proliferate, and the peripheral blood (PB) where they become quiescent. Strong evidence exists for a crucial role of B cell receptor (BCR) triggering, either by (self-)antigen or by receptor auto-engagement in the lymph node (LN) to drive CLL proliferation and provide adhesion. The clinical success of Bruton’s tyrosine kinase (BTK) inhibitors is widely accepted to be based on blockade of the BCR signal. Additional signals in the LN that support CLL survival derive from surrounding cells, such as CD40L-presenting T helper cells, myeloid and stromal cells. It is not quite clear if and to what extent these non-BCR signals contribute to proliferation in situ. *In vitro* BCR triggering, in contrast, leads to low-level activation and does not result in cell division. Various combinations of non-BCR signals delivered via co-stimulatory receptors, Toll-like receptors (TLRs), and/or soluble cytokines are applied, leading to comparatively modest and short-lived CLL proliferation *in vitro*. Thus, an unresolved gap exists between the condition in the patient as we now understand it and applicable knowledge that can be harnessed in the laboratory for future therapeutic applications. Even in this era of targeted drugs, CLL remains largely incurable with frequent relapses and emergence of resistance. Therefore, we require better insight into all aspects of CLL growth and potential rewiring of signaling pathways. We aim here to provide an overview of *in vivo* versus *in vitro* signals involved in CLL proliferation, point out areas of missing knowledge and suggest future directions for research.

## Introduction

CLL is the most frequent hematologic cancer and is characterized by the clonal expansion of CD5^+^CD19^+^ malignant B cells (1). CLL patients can be distinguished into 2 categories with distinct clinical outcome, based on the presence or absence of somatic hypermutation in the immunoglobulin heavy chain variable region (IGHV) genes of the clonotypic B cell receptor (BCR). Patients with low IGHV mutation levels (<2% change from the germline sequence), referred to as unmutated (um-CLL), experience a significantly more aggressive disease than those with mutations, referred to as mutated (m-CLL). IGHV mutation status remains one of the most robust prognostic markers in CLL, yet it does not entirely reflect the heterogeneity of the disease (2).

In addition, CLL is a prime example of a B cell malignancy that is crucially dependent on signals from the microenvironment. CLL cells cycle between lymphoid tissue sites and peripheral blood (PB). CLL cells accumulating in the PB become quiescent, whereas active CLL cells at lymphoid tissue sites are provided with signals from surrounding cells, such as CD40L-presenting T helper cells, myeloid, and stromal cells (3). Since CLL cells are strictly microenvironment-dependent, the crosstalk with the surrounding microenvironment in promoting CLL survival and proliferation has been a focus of intense research.

In order not to perish by neglect, CLL cells need to return to the proliferation sites in lymphoid tissues. This notion is supported by BCR kinase inhibitors that have entered the clinic, foremost the Bruton's tyrosine kinase (BTK) inhibitor ibrutinib, which was found to induce lymphocytosis in patients due to the release of activated CLL cells from lymphoid tissue sites into the PB, preventing migration back into the lymphoid tissue sites and thereby halting disease progression. However, stopping ibrutinib treatment reverses remission and some patients may relapse even on ibrutinib treatment, highlighting the need for greater understanding of the mechanisms that promote CLL proliferation (4).

Strong evidence exists for a crucial role of BCR signaling to drive CLL disease progression, especially the success of inhibitors targeting BCR-associated kinases (5). However, *in vitro* BCR triggering only leads to low-level activation without induction of proliferation, suggesting that additional factors that play a role *in vivo* are missing (6). Several other receptors are known to mediate interactions between CLL cells and the microenvironment, such as CD40 or Toll-like receptor (TLRs), in combination with cytokine receptors which have been shown to induce proliferation upon *in vitro* stimulation (7, 8). It is not quite clear if and to what extent these non-BCR signals contribute to proliferation *in vivo*. Perhaps a combination of stimuli is what may really drive CLL proliferation *in vivo*, or makes CLL develop into more aggressive disease.

In this review, we aim to provide an overview of *in vivo* versus *in vitro* signals involved in CLL proliferation. With focus on BCR, CD40 and TLR signaling, we will attempt to separately describe *in vivo* and *in vitro* data and, in each case, discuss how these receptor-mediated signaling modes may drive CLL. By integrating multiple facets of the CLL microenvironment, we aim to bridge the gap between *in vivo* and vitro studies, point out areas of missing knowledge and suggest future directions for research.

## In Vivo CLL Proliferation

The conceptual framework of CLL biology has changed over the past decades. The traditional view was that CLL is a disease deriving from an inherent defect in apoptosis, in which slowly proliferating CLL cells accumulate due to diminished cell death. In this view, CLL cells continue to accumulate until they reached a level that is detrimental to the patient (9). However, *in vivo* labeling studies using deuterated water have changed this view by documenting the dynamic cellular kinetics of CLL cells. These studies showed that CLL is a dynamic condition, comprising of CLL cells that multiply and die at variable rates (9). Proliferation rates in patients with stable white blood cell count (WBC) indicated that CLL cells are continually dying and replenishing. Therefore, fast clonal birth is not necessarily associated with high WBC increase, suggesting that WBC does not reflect underlying cellular dynamics but rather the net effect of clonal turnover between cell birth and death rates (10). These studies consistently estimated that between 0.07–1.75% of CLL cells circulating in the PB are added to the population each day (9–13). Importantly, patients with birth rates >0.35% were much more likely to exhibit active or progressive disease than patients with lower birth rates (10). Also in patients with recently diagnosed disease, high CLL birth rate was a strong predictor of the need for earlier initial treatment, reinforcing the concept that enhanced cell proliferation is an important driver in the biology of disease progression (13, 14).

Interestingly, both birth rates and death rates of CLL cells were lower than those of healthy B cells, suggesting that CLL cells divide slower and have a lower turnover than their normal counterpart (11, 12). In addition, telomere lengths of CLL cells were shorter than those of healthy B cells in age-matched healthy donors (HDs), showing that CLL cells completed more rounds of proliferation than healthy B cells (15). These observations indicate that CLL progression appears to be the consequence of an imbalance of decreased cell turnover combined with excess proliferation, resulting in a longer replicative history of CLL cells (11).

The observed *in vivo* proliferation rates of CLL cells promote the acquisition of genetic mutations (16). Combined with the fact that CLL cells are less susceptible to apoptosis, CLL cells are able to obtain a more extensive replicative history, suggesting that disease progression is not a result of accumulation but rather of stochastic generation of subclones. Over time, more pathological subclones could be selected which may further affect CLL birth and death rates (9). Importantly, the accumulation of genetic changes may eventually result in subclones that may prevail of microenvironmental control at later stages (17). The insight that CLL is a dynamic disease with both substantial proliferation and death rates is important, since this allows novel clonal variants to expand more quickly to a substantial level (10). Clonal evolution with outgrowth of novel variants harboring genetic alterations has been well described in CLL and has significant impact on clinical outcome (18). However, *in vivo* labeling studies have not managed to link such genetic aberrations to increased proliferation rates (13), suggesting that they are a consequence rather than the cause of increased proliferation in CLL.

### CLL Proliferation Occurs at LN Sites

In aforementioned *in vivo* labeling studies, the fraction of labeled CLL cells in many patients continued to increase for many weeks after the end of the labeling phase, implying they had to have remained in a separate compartment for some time prior to being released into the PB (10). Subsequent *in vivo* labeling studies identified the LN as the anatomical site harboring the largest fraction of newly born CLL cells, with birth rates as high as 3.3% of CLL cells circulating in the PB per day. In contrast, the BM did not seem to be a major proliferation site (12). Gene expression profiling and Ki-67 staining support that active proliferation occurs in the LN from which newborn cells enter the PB (14, 19). Finally, immunohistochemical studies have demonstrated the presence of proliferating CLL cells within specific structures in the LN, resembling proliferation centers, otherwise known as pseudofollicles (20, 21).

**Figure 1:**
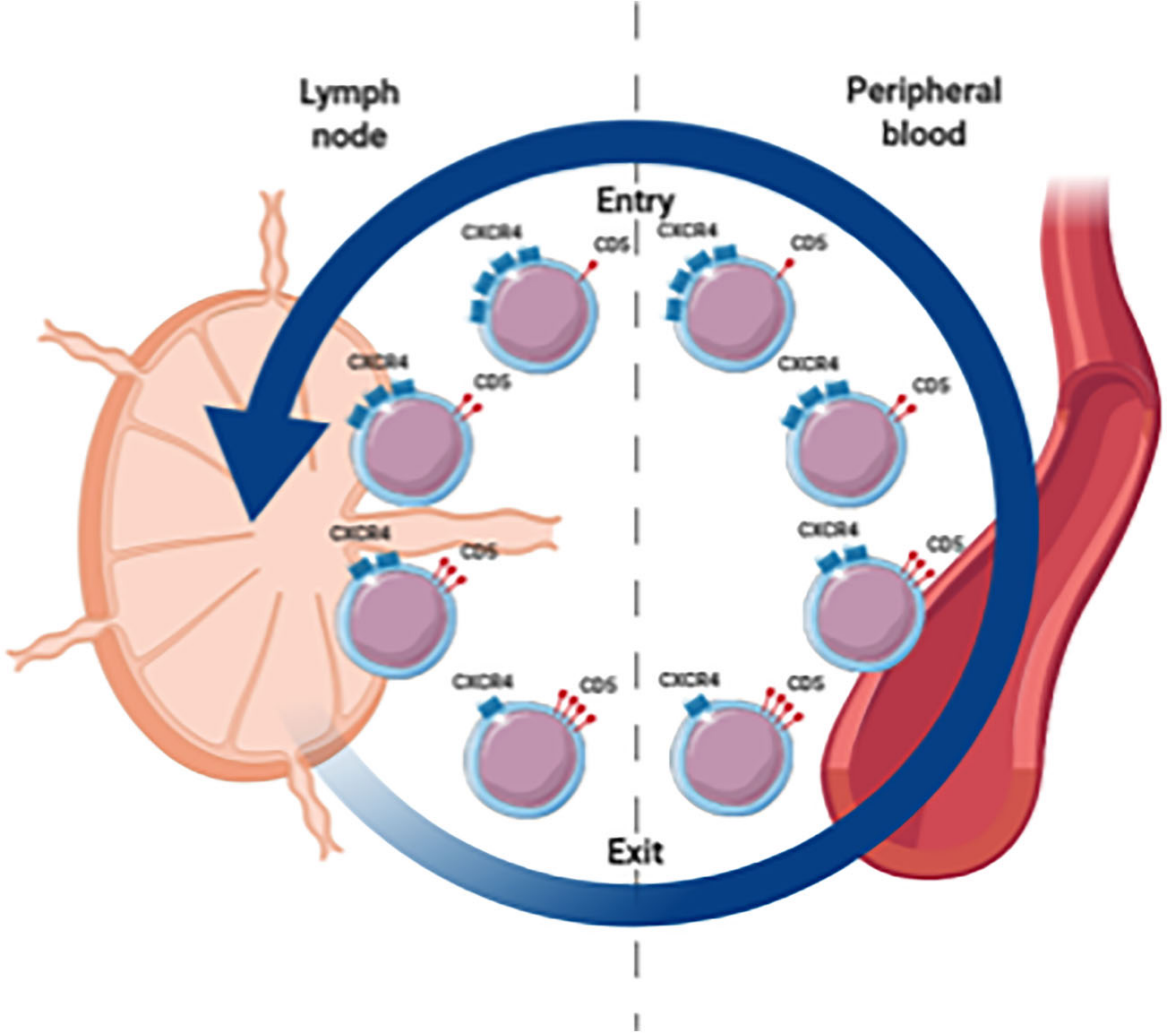
Spectrum of circulating CLL cells illustrating the proliferative fraction enriched in LN emigrants versus the resting fraction enriched in LN immigrants. CXCR4high/CD5low cells are in a resting state. When these cells are activated in the LN, CD5 is upregulated and CXCR4 is internalized. These CXCR4low/CD5high cells are released from the LN and migrate into the PB. Eventually these cells become more quiescent leading to downregulation of CD5 and renewed surface expression of CXCR4 which increases the likelihood of a return to the LN.

Extensive immunophenotyping and intraclonal analyses suggest a spectrum of circulating CLL cells with at one end the proliferative fraction, enriched in recently divided cells that have recently emigrated from the LNs (CXCR4^low^/CD5^high^), and at the other end the resting fraction, enriched in older, less vital cells that need to either immigrate back to the LN or die (CXCR4^high^/CD5^low^) (**Figure 1**) (14, 22). Moreover, gene expression analysis indicated higher levels of pro-proliferation and anti-apoptotic genes in the proliferative CXCR4^low^/CD5^high^ fraction (22).

Thus, LN tissues are the preferred site for CLL cell proliferation, possibly due to accessory cells within the microenvironment that promote proliferation, propagated through diverse receptors such as the BCR, CD40, and TLRs (19, 23, 24). Characterization of BCR, CD40, and TLR signaling in primary CLL cells of the proliferative fraction may pinpoint the importance of each of these individual modes of stimulation and is of interest for understanding the process by which CLL cells residing in these proliferative niches are contributing to disease progression. Aside from BCR, CD40, and TLR stimulation, various other *in vitro* stimulations and culture conditions have been applied in the context of CLL proliferation, including factors like BAFF and APRIL, as well as co-culture with stromal cells, follicular dendritic cells or nurse-like cells (1). These and other candidates are certainly of interest, yet the interaction with feeder cells combined with their secretion of cytokines makes the identification of essential factors difficult (6). In addition, our previous efforts have not managed to show a direct role for BAFF or APRIL in human CLL proliferation (25, 26). Therefore, we take a restricted approach and in the first part of the review we will provide an overview of the *in vivo* evidence of BCR, CD40, and TLR signaling in CLL proliferation, and in the second part of the review we will cover the *in vitro* data that support the role of BCR, CD40, and TLR signaling in the proliferation of CLL.

### In Vivo BCR Signaling

**Figure 2:**
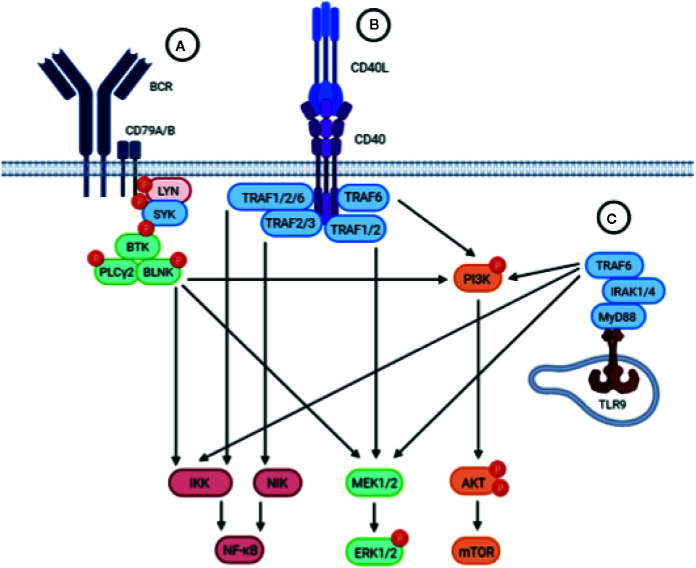
Schematic representation of the BCR, CD40 and TLR signaling pathways. Upstream triggering of BCR, CD40, or TLR signaling lead to activation of similar downstream pathways, including the NF-kB, MEK/ERK, and PI3K/AKT/mTOR pathways. **(A)** The BCR is composed of an antigen-specific surface membrane immunoglobulin paired with the signal transduction component consisting of the CD79A/B heterodimer. Engagement of the BCR by antigen results in aggregation of BCR components that leads to the phosphorylation of ITAMs in the cytoplasmic tails of CD79A/B which triggers the recruitment and activation of the proximal tyrosine kinases LYN and SYK. Subsequently, BTK is activated which will activate PLCγ2 and BLNK, resulting in activation of downstream signaling pathways. **(B)** Upon binding of CD40L, the CD40 receptor on CLL cells trimerizes leading to the recruitment of TRAFs to the cytoplasmic domain of CD40. TRAFs may then cooperate in order to activate different signaling pathways downstream. **(C)** Upon TLR9 activation in the endosomal compartment of CLL cells, the TIR domain of TLR9 engages the TIR domain of the adaptor protein MyD88. MyD88 contains an IRAK1 domain which activates TRAF6, leading to the activation of downstream pathways.

Signaling through the BCR pathway is a key functional step of all normal and malignant B cells and is also a critical component in CLL (27). BCR signaling activity is elevated in CLL cells compared to healthy B cells, and deregulated BCR signaling is considered a driving mechanism leading to CLL development, progression, and relapse (28). BCR triggering leads to the activation of downstream signaling pathways, including the MAPK/ERK, PI3K/AKT/mTOR, and NF-κB pathways, which play a role in CLL survival and proliferation (**Figure 2**) (5, 29–31).

Critical evidence of the involvement of BCR stimulation in driving CLL is the expression of BCR-associated genes in LN CLL cells (19). In addition, LN CLL cells had higher pSYK levels compared to CLL cells from PB or BM, supporting BCR-dependent activation of CLL cells *in vivo* and suggesting that the LN is the crucial site for proliferation (19). Mouse models exhibiting spontaneous CLL development also show an important role for BCR signaling in the onset and development of CLL. These studies showed that overexpression of BTK leads to accelerated CLL onset (32). The IgH.ETµ mouse model shows that BTK expression is a prerequisite for CLL development (32) whereas the TCL1 mouse model showed that mice with ablation of BTK significantly delayed CLL development but still developed leukemia at rates similar to wild type TCL1 mice treated with ibrutinib (33–35).

In patients, CLL cells have elevated BTK expression (36) and pBTK levels compared to healthy B cells (28), as well as lower expression of surface IgM (sIgM) (37) which is additional evidence for BCR stimulation *in vivo*, as sIgM expression in CLL cells is downregulated after antigen stimulation which is reversed during circulation in the PB (38). This observation suggests endocytosis of sIgM *in vivo* which can only be due to interaction of the BCR with a ligand able to bind (27, 39). In CLL cells isolated from the PB, subgroups of cells could be distinguished with increasing sIgM and CXCR4 expression, likely regulating the ability to migrate to the LN and engage antigen in situ (38).

However, this raises questions in the context of the two CLL subsets stratified by IGHV gene mutations. Specifically, as um-CLL cells have polyreactive BCRs that may respond to a wide spectrum of epitopes whereas m-CLL cells have undergone somatic hypermutation and thus express BCRs specific to a certain epitope, and may therefore be less responsive to external signals (5, 40). Indeed, *in vivo* labeling studies measured higher CLL growth rates in the LNs of um-CLL patients compared to m-CLL patients (14). Consistent is the finding that um-CLL cells had much shorter telomeres than m-CLL cells, implying a more extensive replicative history (15).

Furthermore, LN CLL cells showed increased BCR signaling in um-CLL compared to m-CLL (19). This is consistent with findings in PB CLL cells, where even though BCR signaling was significantly lower, a preferential expression of BCR-regulated genes was found in um-CLL as compared to m-CLL, which most likely reflects BCR activation in the LN as cells carry a temporary imprint of their prior stimulation (19, 41). However, even in m-CLL patients, BCR signaling in LN CLL cells was significantly higher compared to PB, indicating that BCR signaling is also involved in this subset of CLL (4). ZAP-70 expression, which is one of the most prominent genes distinguishing um-CLL from m-CLL, may reinforce BCR responsiveness (42). ZAP-70 is structurally similar to the BCR-associated kinase SYK (5) and its expression was associated with greater BCR signaling capacity, implicating a role for the BCR in CLL proliferation (43). Therefore, this suggests that the ability to respond to antigen stimulation coupled to signal reinforcement may underlie the differences in disease activity between the two prognostic subsets (29, 39, 44–46).

### Antigen-Dependent BCR Signaling

BCR signaling can be broadly divided into two main types: one that is mediated by antigen and another that is independent of antigen, referred to as tonic BCR signalling (1, 47, 48). Antigen-dependent and antigen-independent BCR stimulation are two fundamentally different mechanisms of BCR signaling which exist in B cell lymphomas. However, it is not quite clear to what extent tonic or antigen-dependent BCR signaling play a role in driving CLL. The absence of mutations in BCR signaling components leading to antigen-independent pathway activation in CLL favors a dominant role for antigen-dependent BCR signalling (5). The fact that CLL proliferation only takes place in lymphoid tissues may suggest that relevant antigens are localized to discrete anatomic compartments, but more likely this indicates the lack of additional signals outside of these compartments that trigger CLL proliferation, such as T cell-derived signals in the LN (16).

Strong molecular evidence for antigen-dependent BCR signaling in CLL is the presence of stereotyped BCRs, which support the idea of a common selecting antigenic epitope (49, 50). A study using antigen-specific TCL1 mice showed that neither acute nor chronic exposure to specific antigen influenced disease progression. Rather, CLL clones preferentially selected light chains paired with the antigen-specific heavy chains that conferred CLL cells the ability to interact with a broad range of autoantigens (51). These results suggest that pathogens may drive CLL by selecting and expanding pathogen-specific B cells that cross-react with one or more self-antigens, indicating that the BCR may in fact shape CLL progression *in vivo*. The specific antigens recognized by these stereotyped CLL BCRs are not well described, especially in the case of m-CLL. Whereas m-CLL clones exhibit more restricted autoreactivity (9, 40), the majority of um-CLL clones express low-affinity BCRs that are polyreactive recognizing self-antigens such as DNA, insulin and the cytoskeletal proteins myosin and vimentin, as well as foreign antigens such as bacterial DNA and lipopolysaccharides in addition to a spectrum of molecular motifs exposed on apoptotic cells (40, 52–57). One study showed and identified specificity to an autoantigenic target in one-fourth of CLL cases independent of IGHV mutation status (58). All identified BCR targets were cytoplasmic proteins. The translocation of cytoplasmic antigens to surface membrane blebs and apoptotic bodies would enable binding to the surface BCR of CLL cells (52). Importantly, the same study showed that BCRs belonging to the same stereotyped subset target identical antigens, but surprisingly, BCRs from individual CLL patients were specific for different epitopes of the same antigens. Finally, binding of the autoantigen to the respective CLL cells induced specific activation and proliferation, suggesting that autoreactivity of CLL cells *via* the BCR may be a general mechanism for driving CLL. Other stereotypic subsets of m-CLL have been described showing specificities to the cytomegalovirus phosphoprotein pUL32, the Fc-tail of IgG, as well as specificity to β-(1,6)-glucan, an abundant component in yeast and filamentous fungi (50, 59–61). However, it should be noted that there have been no recent reports of new subsets exhibiting specificity to exogenous antigens, illustrating that perhaps this represents a unique attribute of only a few CLL subsets. A study with TCL1 mice expressing transgenic BCRs with different antigen specificities showed that chronic interactions with low-affinity can induce CLL *in vivo*, whereas interactions with high-affinity antigens cannot (62). Additionally, the authors showed that low-affinity BCRs are positively selected, whereas high-affinity BCRs are not. Consequently, m-CLL clones remain more stable overall and expand at a slower rate, likely due to high-affinity binding to restricted sets of antigenic epitopes that either occur infrequently because they are on foreign antigens or because they induce anergy due to high-affinity binding (5).

Anergy is one of the mechanisms of the immune system to silence autoreactive B cells upon recognition of self-antigens. A state of BCR desensitization is induced by chronic binding of antigen *in vivo*, resulting in unresponsiveness when cells are stimulated with antigen *in vitro*. The fact that m-CLL is associated with a favorable disease course and bind antigen more specifically with higher affinity than um-CLL, suggests that exposure to antigen *in vivo* may lead to anergy of CLL cells (63). In mouse models, anergic B cells showed features of low levels of sIgM as the result of constant BCR internalization, increased basal intracellular calcium concentrations and constitutive activation of ERK1/2. This biochemical program is reversible and lasts as long as B cells are exposed to the antigen (64). Notably, repetitive BCR stimulation in healthy B cells resulted in anergy and CD5 expression, which is a feature of CLL (65). In addition, mouse models have shown that CD5 is necessary to maintain anergy in B cells as knocking out CD5 in these mice resulted in a loss of self-tolerance (66). In vitro studies analyzing responses to BCR triggering have identified a subset of CLL patients exhibiting indolent disease with CLL cells containing anergic features (67), including a lack of BCR signaling capacity and constitutive activation of ERK1/2 (64). Additionally, the BCR-associated kinase LYN initiates a negative feedback loop *via* recruitment of the phosphatase SHP-1 which inhibits BCR signaling and is overexpressed in CLL, further illustrating an anergic phenotype (68). Several studies have shown that the anergic phenotype of CLL cells, including sIgM expression and signaling capacity, reverses spontaneously after culture *in vitro* or following capping and endocytosis (39). This shows that downstream signaling pathways appear to be intact and that anergy can thus be attributed to surface immunoglobulins (sIgs), and this also provides direct evidence for engagement of putative antigen *in vivo* (39, 64).

### Antigen-Independent BCR Signaling

The lack of antigen reactivity in m-CLL may indicate a role for antigen-independent BCR signaling, which is supported by the observation of phosphorylated LYN, SYK and canonical NF-κB in unstimulated CLL cells (5). Ligand-independent signaling is frequent in other malignancies where proliferation is subverted by the acquisition of genetic mutations in signaling components mimicking physiological stimulation of receptors (69). Such mutations in BCR signaling components are absent in CLL (5).

A special type of antigen-independent BCR activation has been described in CLL, which involves the binding of auto-epitopes existing on adjacent or in the same sIgs. Reactivity with sIg auto-epitopes could occur on a continuous basis. This also suggests a mutual BCR recognition on CLL cells which was confirmed by the binding of secreted CLL-derived BCRs to the surface of cell lines expressing either CLL or HD BCRs, but not to cells that did not express a BCR (70). Likewise, serum immunoglobulins from HD plasma were not able to bind HD B cells whereas serum immunoglobulins from CLL plasma were (71). It was shown that the binding was mediated by a conserved epitope in the second framework region of VH domains of the CLL BCR, as point mutations inside this motif abolished autonomous signaling. This motif was located outside of the antigen-recognition site, indicating that the induced signaling is not mediated by external antigens. Consistently, VH domain point mutations of antigen-specific BCRs in m-CLL were still able to signal upon recognition of antigen, indicating that this type of tonic signaling is antigen-independent but does not rule out the involvement of extrinsic antigens in the pathogenesis of CLL (70). Consistent with these observations are imaging data showing that HD BCRs exist predominantly as monomers and dimers in the plasma membranes of resting B cells and form oligomeric clusters upon stimulation with antigen. In contrast, CLL BCRs form dimers and oligomers in the absence of a stimulus, reflecting an antigen-independent tonic activity of the BCR (72). A single amino acid exchange reverted the organization to monomers and thus prevented auto-aggregation of CLL BCRs (72).

As tonic signaling would essentially result in constitutive BCR signaling, this would thus lead to a tolerogenic signal that should result in anergy (5). Consistently, a lack of external BCR stimulation does not lead to spontaneous CLL proliferation *in vitro* (5). Therefore it can be hypothesized that binding of extrinsic cognate antigens is essential to overcome anergy of CLL cells and is thus required for CLL proliferation (50, 73). A study using the TCL1 transgenic mouse model showed that this unique autonomous signaling capacity is a prerequisite for CLL development. Moreover, the capacity of CLL cells to respond to antigen inversely correlated with time to leukemia development, suggesting that signals induced by external antigens contribute to the aggressiveness of the disease (62).

In summary, both antigen-dependent and antigen-independent BCR signaling have been described in CLL, and CLL cells can receive both continuous and intermittent BCR signals that may facilitate proliferation (5). Yet, ligand-dependent and tonic BCR signaling may not be mutually exclusive. CLL clones could originate as antigen-dependent, but evolve to become more autonomous if the critical BCR regions are mutated. Substantiating this possibility would require comparison of BCR sequences in a cohort containing early MBL and later CLL stages of the same patient, which to our knowledge has not been performed. Alternatively, these separate mechanisms could reflect different routes for clonal expansion after initial transformation (69).

### BCR Inhibitors

The BCR signaling pathway has emerged as an important therapeutic target for B cell malignancies, including CLL (74). Several BCR-targeted agents, including inhibitors of BTK (ibrutinib), PI3Kδ (idelalisib), and SYK (fostamatinib) have demonstrated clinical efficacy, which led to FDA approval of idelalisib and ibrutinib (28, 75–78). Especially the introduction of ibrutinib has dramatically changed the management of CLL, allowing for treatment without chemotherapy (74). Ibrutinib inhibits the activation of BTK, which plays a role in proliferation, survival, migration and tissue adhesion of CLL cells (4, 79, 80). Treatment of patients with ibrutinib leads to lymphocytosis due to efflux of CLL cells from the proliferative LN compartment into the PB (28, 81), thereby depriving CLL cells from microenvironmental signals and halting disease progression (17).

The impact of ibrutinib treatment on *in vivo* CLL kinetics of CLL cells showed that no newly divided CLL cells entered the PB upon ibrutinib treatment. The average pretreatment birth rate decreased upon ibrutinib treatment whereas death rates increased (81). In addition, ibrutinib treatment resulted in a reduction of the proliferative CXCR4^low^/CD5^high^ fraction (82). Even though ibrutinib targets a key pathogenic pillar of CLL by depriving cells from antigen and interactions with the lymphoid microenvironment, it is not sufficient to eradicate disease, as stopping treatment reverses remission (17). The fact that the BCR components BTK and PLCG2 are specifically mutated in ibrutinib-resistant CLL underlines that therapeutic success depends critically on inhibition of this pathway (83).

Importantly, it was shown that maximum inhibition of BCR signaling *in vivo* was already achieved after one administration of the drug whereas maximum inhibition of downstream NF-κB signaling required repeated dosing (4). This indicates that aside from direct effects, continued treatment of ibrutinib leads to changes in the microenvironment that have indirect effects on CLL cells, highlighting the role of accessory cells mediating signaling *via* alternative receptors such as CD40 and TLRs. Next, we will discuss the existing evidence for *in vivo* CD40 and TLR signaling and their roles in CLL proliferation.

## Evidence for Non-BCR Signals In Vivo

### In Vivo CD40 Signaling

CD40 expressed on CLL cells can be stimulated by its physiological ligand CD154 (CD40L) expressed on, for instance, activated CD4 T cells and follicular T helper cells (84, 85). Interaction of CD40 and CD40L stimulates the proliferation and differentiation of healthy B cells (85). CD40 stimulation on CLL cells activates downstream signaling pathways including MAPK/ERK, PI3K/AKT/mTOR, and NF-κB (86), thus largely overlapping with downstream BCR signaling pathways (**Figure 2**). As a result, CLL cells are activated and provided with strong survival signals rendering them highly resistant to a wide variety of therapeutics (7, 85). In addition, CD40 stimulation propels both CLL cells and healthy B cells in a proliferative state (7, 87). Interestingly, a few studies have reported the expression of CD40L on CLL cells as well, suggesting a mechanism in which activated CLL cells may directly stimulate CD40 on resting bystander CLL cells in a paracrine manner (88, 89).

The earliest observation for a role of CD40 signaling in CLL was the infiltration of CD4 T cells that express CD40L in CLL pseudofollicles that co-localize with Ki-67^+^ CLL cells in these proliferation centers (23, 90). This is suggested to be a mechanism to regulate CLL proliferation, which was supported by *in vitro* stimulation of PB CLL cells with CD40L, inducing expression of CCL22, which serves as an attractant for CD4 T cells which in turn express CD40L (23). Moreover, co-culture of CLL cells with activated autologous T cells results in proliferation of CLL cells (7, 91). Importantly, interference with CD40 signaling collapses LN germinal centers necessary for B cell development, differentiation and somatic hypermutation (92).

Interestingly, in lymphocytes isolated from PB of CLL patients, a fraction of proliferating T helper cells were observed in the presence, but not in the absence of CLL cells. Moreover, these CLL-associated T helper cells induce HLA class II-dependent activation and proliferation of CLL cells *in vitro*, suggesting that CLL is a disease driven by immune responses *via* a process in which T helper cells engage CLL cells in response to antigen presented on the CLL cells’ own HLA class II molecules (93). It would be worthwhile if these intriguing findings can be confirmed by other studies.

Even though direct evidence for T cell-dependent CLL proliferation in patients is lacking, several mouse models have provided more insight. CLL cells xenografted in NOD-SCID mice require activated autologous T cells in order for the CLL cells to proliferate. Moreover, depletion of CD4 T cells inhibited proliferation whereas depletion of CD8 T cells did not (94). Also, LMP1/CD40 mice express a chimeric protein containing part of the Epstein-Barr viral Latent Membrane Protein 1, mimicking constitutively active CD40 triggering (95). These mice showed an increase of B cells in secondary lymphoid organs with an activated phenotype, increased proliferation and prolonged survival. In addition, they showed significantly impaired T cell-dependent immune responses, thus resembling CLL in many aspects (96). Moreover, LMP1/CD40 cells proliferated spontaneously *in vitro* in a CD40-dependent manner (95).

Combined, these observations indicate that CD40 may in fact be an important mediator in CLL proliferation, which is currently less widely recognized compared to the contribution of BCR signaling.

### In Vivo TLR Signaling

Recurrent mutations in CLL include MYD88, a gene which encodes a downstream component of TLR signaling (69). TLRs are part of the innate immune system and respond to specific molecular patterns found in various microorganisms, including bacteria (43). These receptors are expressed in CLL cells and biologically active, suggesting an additional route of stimulation besides BCR signaling (69). TLR signaling leads to the activation of several downstream signaling pathways, including MAPK/ERK, PI3K/AKT/mTOR, and NF-κB (97), resulting in the activation and proliferation of CLL cells (43) (**Figure 2**).

The most evident observations for a role of TLR signaling in CLL are increased expression of TLR9 in CLL cells compared to healthy B cells (98, 99), TLR pathway activation in LN CLL cells as shown by gene array studies (19, 24), as well as *in situ* proximity ligation assay experiments that showed the interaction of pIκBα with TLR9 and MYD88 in LN CLL cells (24). CLL BCR specificity for DNA or antigens physically linked to DNA further suggest a role for TLR signaling in driving CLL (8). Moreover, apoptosis-associated antigens bound by sIgs can also be recognized by TLRs after entrance to the endosomal compartment *via* sIgs (52). Interestingly, the observation that um-CLL cells are more responsive to BCR activation than m-CLL cells is mirrored by TLR activation *in vivo* and *in vitro* (24). A possible explanation is that the antigen-specific BCRs in m-CLL make them less likely to internalize antigen-linked TLR ligands compared to um-CLL, whose BCRs are polyreactive and bind with low affinity to a wide variety of antigens (52, 54, 100). Yet, this does not hold for CpG stimulation *in vitro*, as these are internalized in a BCR-independent fashion. See below in the section *in vitro TLR signaling* for additional observations that TLR and BCR signaling may cooperate to promote CLL proliferation in um-CLL.

The absence of TIR8, a negative regulator of TLR signaling, was shown to accelerate disease progression in TCL1 mice (101). Moreover, epidemiological studies found an increased risk for the development of CLL following episodes of sinusitis, pharyngitis, influenza, cellulitis, and herpes zoster, where risk increased with increasing severity or frequency of infection (102–104). These studies suggest that infectious agents can promote disease onset and progression of CLL, which may be related to TLR activation (102).

It is not yet clear to what extent these non-BCR signals contribute to proliferation in situ, but it is apparent that both BCR, CD40, and TLR activation all show marked similarities in the downstream signaling pathways involved, including the MAPK/ERK, PI3K/AKT/mTOR, and NF-κB pathways (43) (**Figure 2**). Perhaps a combination of stimuli is what may really drive CLL proliferation *in vivo* or helps develop it into more aggressive disease.

### In Vitro CLL Proliferation

Despite CLL being a proliferative disease with significant cell turnover, primary CLL cells rapidly undergo apoptosis in the absence of microenvironmental survival signals (105). What further illustrates that essential *in vivo* factors are missing in *in vitro* systems, is that co-culture with stromal cells or the addition of soluble factors can significantly extend CLL survival, yet only for a limited amount of time and thus far, no system permits the long-term expansion of CLL cells *in vitro* (7, 19, 94, 106, 107). In vitro studies typically analyze CLL cells isolated from PB and consequently, the contribution of the host microenvironment to the proliferation of CLL cells *in vivo* remains ill-defined (19). The difficulties of mimicking a physiologic microenvironment supporting CLL proliferation hinder *in vitro* studies and as a result, a large variety of culture systems have been developed in order to investigate CLL proliferation (105). This raises difficulties in comparing data achieved with these highly variable approaches and hinders systematic characterization of culture systems or back-to-back comparisons in terms of CLL proliferation (105, 106). In the next section, we will elaborate on CLL proliferation in the light of *in vitro* data and we will specifically discuss models utilizing BCR, CD40 or TLR stimulation in combination with costimulatory cytokine signals.

### In Vitro BCR Signaling

Despite the consensus regarding the role of BCR signaling in the biology of CLL, the response of CLL cells to BCR stimulation *in vitro* is notoriously heterogeneous among patient samples (43). In vitro BCR stimulation of CLL cells is performed using anti-IgM, either soluble or immobilized. In vitro responses to BCR stimulation differ between um-CLL and m-CLL as demonstrated by several groups using multiple assays, including global protein tyrosine phosphorylation, gene expression profiling, cellular metabolic activity, apoptotic response and proliferative activity (29–31). Several studies have reported that response to BCR stimulation was correlated with sIgM levels (31). Some studies found higher sIgM levels in um-CLL (108), whereas others found little to no differences between um-CLL and m-CLL (39, 46), claiming a role for additional factors contributing to BCR responsiveness (39). Many studies showed a correlation of CD38 and ZAP-70 expression with BCR responsiveness, however, CD38 does not influence BCR signaling *in vitro* and ZAP-70 is not required for response. Therefore, these correlations most likely do not result from functional interactions, but are a result of um-CLL expressing higher levels of CD38 and ZAP-70 (39). Some discrepancies found in literature concerning *in vitro* responses to BCR stimulation can, at least partially, be attributed to the lack of a standardized protocol to trigger the CLL BCR *in vitro* (109). For example, it has been shown that immobilized anti-IgM provides a more potent *in vitro* CLL stimulus than soluble anti-IgM (109, 110).

Another possibility is that the variability in BCR responses stems from variable degrees of sIg clustering, which may be associated with natural genomic heterogeneity in BCRs and/or response to antigen (71). Indeed, induction of stable BCR clustering on healthy B cells modulated BCR responsiveness. In fact, by titrating the amount of anti-IgM crosslinking, healthy B cells could be induced to recapitulate the diversity in signaling observed in CLL cells, confirming that BCR clustering can modulate BCR responsiveness and thereby phenocopy the signaling dysfunction in CLL (71). As for the heterogeneity of BCR responsiveness, CLL cells could be divided into 3 subgroups of SHP-1^low^/pPLCG2^high^ to SHP-1^high^/pPLCG2^low^ expression, where each subset displayed unique deviations in their BCR signaling responses (71). As increasing levels of SHP-1 and decreasing levels of pPLCG2 correlated with weakened BCR responsiveness, this suggests that phenotypic variability within isogenic populations of cells may result from heterogeneous levels of signaling regulators (71).

Whereas BCR stimulation of healthy B cells significantly induces their proliferation, *in vitro* BCR stimulation of CLL cells does not lead to proliferation (107), which is another reminder that the *in vitro* CLL systems are missing a crucial aspect that is active in patients. In vitro engagement of the BCR in CLL promotes G1 cell cycle progression as shown by increased levels of cyclin D2 and CDK4, but does not induce cell division, associated with constitutively high levels of the cell cycle inhibitor p27 (30). Accordingly, CLL cells within proliferation centers of the LN showed high expression of cyclin D2 and downregulation of p27 (111). Therefore, CLL proliferation may depend on costimulatory signals such as those delivered through CD40 or TLRs, possibly in combination with cytokines (6, 7, 16). The interleukins are a family of cytokines that serve as key regulatory elements within the immune system and a number of specific interleukins have been identified as being associated with the proliferation of CLL cells *in vitro* (43). Importantly, IL-4 promotes the expression and function of surface IgM in CLL cells, thereby enhancing *in vitro* BCR responsiveness (112). However, proliferation of CLL cells has been mostly described in an antigen-independent context using combined stimulations with CD40L/IL-21, CD40L/CpG, or CpG/IL-15 (107), which we will describe in the next sections.

### In Vitro CD40 Signaling

In vitro CD40 stimulation of CLL cells can be performed using either soluble agonists or *via* co-culture with CD40L-presenting cells. Importantly, soluble agonists like anti-CD40 antibodies or soluble CD40L are inferior to co-culture with CD40L-presenting cell lines which are able to support proliferation more efficiently (85, 113, 114). Again, as various soluble CD40 agonists and CD40L co-culture systems are used, this hinders direct comparison of *in vitro* studies on CD40-mediated CLL proliferation (85).

Similar to BCR stimulation, crosslinking of CD40 provides only weak proliferative responses in CLL whereas healthy B cells proliferate well (87). In contrast to single BCR or CD40 stimulation, co-culture of CLL cells with activated CD4 T cells promoted CLL proliferation to the same extent of that in healthy B cells, which respond equally well to single CD40 stimulation or co-culture with T cells (87, 93). Although a monolayer of CD40L-presenting fibroblasts induced a highly similar gene profile as induced by co-culture with T cells (7), it did not induce CLL proliferation like activated T cells (106), highlighting the role of additional T cell-derived signals in the proliferation of CLL.

CD40 triggering upregulates the IL-21 receptor, making CLL cells more receptive for the T cell cytokine IL-21, which has been implicated in CLL proliferation (115). IL-21 significantly increased CD40-mediated proliferation, and CLL proliferation by activated T cells was shown to be IL-21-dependent as well (7, 116). Importantly, *in vitro* T cell activation induced IL-21 mRNA production, specifically in follicular T helper cells, which have been shown to be present and produce IL-21 in LNs *in vivo* (7, 117). Another T cell cytokine implicated in CD40-mediated CLL proliferation is IL-4. In vitro stimulation with soluble CD40L caused a slight increase of CD40 expression on CLL cells but stimulation with IL-4 resulted in a significant increase of CD40 expression (118). Combined stimulation of CD40 and IL-4 however results in only modest proliferation of CLL cells (116), but it primes cells for proliferation *via* IL-21, as combined stimulation of CD40L, IL-4 and IL-21 results in increased CLL proliferation (117).

**Figure 3:**
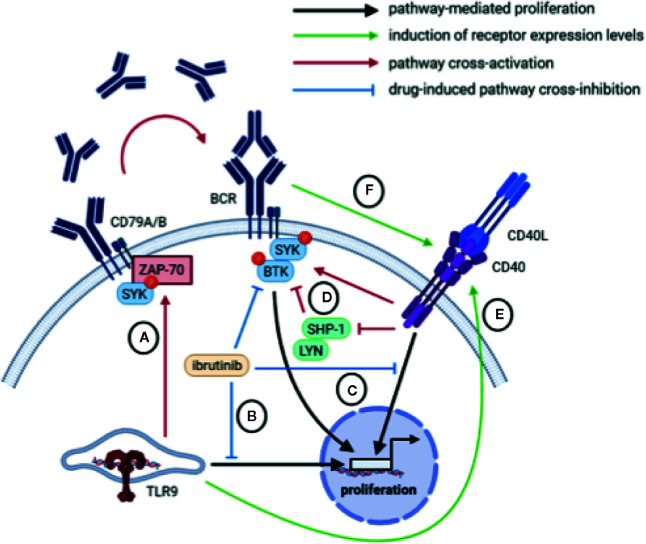
Overview of potential crosstalk mechanisms between BCR, CD40, and TLR signaling. **(A)** TLR signaling in um-CLL may mount an autocrine feedback loop mediated by ZAP-70 and SYK involving the production and secretion of IgM which may subsequently trigger BCR signaling in an autocrine/paracrine manner. **(B)** Inhibition of BCR signaling *via* BTK inhibits CpG-mediated proliferation. **(C)** Inhibition of the BCR-associated kinases BTK or SYK inhibits CD40-mediated proliferation. **(D)** Upon CD40 stimulation, the BCR-associated kinases BTK and SYK are activated, possibly *via* inhibition of negative feedback components LYN and SHP-1. **(E)** TLR9 stimulation *via* CpG increases expression of the CD40 receptor. **(F)** Activation of BCR signaling increases expression of the CD40 receptor.

Interestingly, *in vitro* BCR stimulation of CLL cells also resulted in increased expression of CD40, suggesting potential crosstalk between BCR and CD40 signaling (31, 93). In fact, whereas BCR stimulation rapidly activates pSYK in both healthy B cells and CLL cells, stimulation with CD40L also activates pSYK in CLL cells but has no effect on pSYK in healthy B cells (119). Likewise, inhibition of SYK hampers CD40-mediated proliferation of CLL cells but not in healthy B cells. In addition to SYK, BTK is also activated upon CD40 stimulation, suggesting that CLL cells exploit CD40 stimulation by increasing BCR pathway activity (119). A possible explanation may be that CD40 acts as a gatekeeper for BCR signaling by inhibiting negative feedback components like LYN and SHP-1, so that CD40-dependent activation of the BCR pathway is required to overcome negative feedback signals in anergic CLL cells (119) (**Figure 3**).

CD40 stimulation in combination with IL-21 or IL-4 is sufficient to induce CLL proliferation *in vitro* whereas for BCR stimulation this is not the case (6, 7). However, CLL cells treated with ibrutinib *in vivo* did not proliferate upon CD40/IL-21 stimulation *in vitro*, an effect which can be recapitulated by *in vitro* inhibition of either BTK, PI3K or SYK, indicating the requirement of BCR kinases in CD40-mediated proliferation (107). CLL proliferation is significantly increased further when BCR stimulation is added to the combined stimulation of CD40 and cytokines whereas for healthy B cells this is not the case, showing that these separate signaling nodes may potentially cooperate to drive CLL proliferation *in vivo* (6).

### In Vitro TLR Signaling

In vitro activation of TLR signaling is carried out using CpG, resulting in endocytosis and subsequent binding of TLR9 in the endosomal compartment, thereby mimicking the interaction of CLL cells with bacteria (120). In vitro stimulation with CpG activates p-p65 and decreases IRAK1 levels, recapitulating the effects of TLR activation as observed in LN CLL cells *in vivo* (24). In vitro stimulation of CLL cells with CpG resulted in quite variable reports of proliferation (99, 107, 121, 122). Similar to *in vitro* BCR activation, um-CLL cells can more often be induced to proliferate upon CpG stimulation (97, 123). Importantly, response is not correlated with TLR9 mRNA or protein expression (97). The *in vitro* proliferative response to CpG was found to be highly predictive of progression-free survival, time to treatment and overall survival in m-CLL, whereas prognosis of um-CLL was equally worse, with or without proliferative response to CpG *in vitro* (124). Although we still do not fully understand the mechanistic basis of TLR signaling in CLL proliferation, these findings do support the relevance of TLR signaling in driving CLL.

In vitro CpG stimulation was shown to upregulate CD122, which is a shared subunit of the receptor for IL-2 and IL-15 (125). Consistently, addition of the T cell cytokine IL-2 significantly enhances CpG-mediated proliferation of CLL cells (7). IL-15, produced by monocytes, synergistically promotes CpG-mediated CLL proliferation independent of CLL mutation status, thus reversing the difference as usually seen between um-CLL and m-CLL (8, 107). Moreover, *in vitro* proliferation mediated by CpG/IL-15 could not be linked to prior treatment or *in vivo* growth rates. As for genetic abnormalities, only TRI-12 was associated with a significantly greater propensity for proliferation in response to CpG/IL-15 (8). Examples of robust *in vitro* proliferation of clones with slow *in vivo* growth rates support the notion that the availability of stimulatory signals within the *in vivo* microenvironment may be as relevant as a cell’s intrinsic potential for proliferation (8). Notably, the abundant presence of IL-15-expressing cells in LNs of CLL patients makes this mechanism clinically relevant (125). Similar to CD40 stimulation, CpG induces upregulation of the IL-21 receptor (126). Whereas healthy B cells proliferated much more than CLL cells following single CpG stimulation, addition of IL-21 rescued enhanced the proliferative activity of CLL cells to the same level of healthy B cells (123).

Links between TLR and BCR signaling have also been described, as for example *in vitro* stimulation of CLL cells with CpG induced phosphorylation of CD79A, LYN and SYK which appears to rely on the expression of ZAP-70 (127). The strong association between CpG-mediated CLL proliferation and IGHV mutation status may suggest that TLR stimulation is modulated by BCR signaling (97, 123). For example, anergic cells that are usually m-CLL and lack expression of ZAP-70, show reduced proliferative responses to CpG *in vitro* (124). Studies have shown that engagement of TLR signaling in CLL is able to mount an autocrine feedback loop involving the production and secretion of IgM leading to activation of the cell’s own BCR, which reinforces the concept of tonic BCR signaling in the absence of antigen (107, 127) (**Figure 3**).

Similar to *in vitro* BCR activation, stimulation with CpG also caused upregulation of CD40 on CLL cells, showing links of TLR stimulation with CD40 signaling as well (99, 122, 128, 129). Activation of TLR9 *via* CpG significantly increased CLL proliferation when combined with CD40 stimulation, similar to CD40/IL-21 stimulation (6, 7, 128, 130). Importantly, combined TLR/CD40 stimulation overcomes the hyporesponsiveness to CpG as often seen in m-CLL (130). On the contrary, CLL cells that do not proliferate *in vitro* in a T-cell dependent manner, can be triggered to proliferate upon addition of CpG/IL-2 (93).

Finally, inhibition of BCR signaling *via* treatment with ibrutinib or *via* inhibition of SYK significantly inhibits both CD40- and CpG-mediated CLL proliferation *in vitro*, showing the role of BCR-associated kinases (36, 107, 131). CD40 and CpG-induced proliferation do however differ in their involvement of the BCR complex. CD40 involves the recruitment of BTK independent of upstream BCR components whereas CpG indirectly triggers the BCR *via* IgM secretion (107). Therefore, BCR-targeted agents effectively target the aforementioned TLR-BCR feedback loop (107).

Concerning the potential cooperation of signaling pathways, it is important to note that a divergence exists between crosstalk of separate pathways and active rewiring of distinct signaling pathways. For example, in diffuse large B cell lymphoma it was found that the GC and ABC subsets depended on the BCR subunits CD79A/B, but engaged divergent downstream signaling pathways (132). In patients with MYD88 mutations, a new complex consisting of TLR9 and MYD88 was found, revealing interactions with the BCR subunits CD79A/B. It was shown that TLR and BCR signaling cooperate to assemble MYD88 in a signalosome which activates mTOR and NF-κB signaling, providing a mechanistic insight as to why these patients were particularly sensitive to the BCR-targeted agent ibrutinib. This same rewiring was not found in CLL samples, but the expression of ZAP-70 represents another example where single activation of TLR9 is sufficient to fully engage BCR signaling (127, 132). ZAP-70 therefore represents an important candidate for signaling pathway rewiring in CLL. It was shown that TLR-mediated BCR activation was not dependent on the kinase activity of ZAP-70, which is compatible with ZAP-70 functioning as a scaffold in a signaling complex that relays TLR9 signals to SYK, thereby integrating innate into adaptive immune responses (42, 127). However, studying rewiring of signaling pathways is difficult and usually requires techniques such as mass spectrometry or proximity ligation assays to study the interactome of proteins, as ultimately protein-protein interactions assemble and regulate signaling pathways (133). Finally, multiomic analyses allow to study changes in signaling networks under specific conditions, and a novel kinomics approach applied in CLL revealed that rewiring of signaling pathways is not strictly oncogenic but can also be influenced by therapy (134). Comparison of kinase fingerprints between treatment-naïve patients and patients who had undergone prior chemoimmunotherapy, revealed SYK as a critical kinase to be differentially active upon BCR stimulation which correlated with proliferative capacity *in vitro* (134).

In summary, various crosstalk mechanisms between BCR, CD40, and TLR signaling have been described in CLL based on receptor expression levels, the activation of downstream mediators as well as the use of targeted inhibitors (**Figure 3**).

## Summary and Outlook

In summary, most evidence for driving CLL proliferation *in vivo* is currently attributed to BCR engagement. A major discrepancy is that BCR stimulation *in vitro* does not induce proliferation, indicating that BCR-induced CLL proliferation *in vivo* likely requires additional (costimulatory) signals that are missing *in vitro*. We have highlighted the role of T cells in the proliferation of CLL cells *in vivo*, as T cell-derived signals including CD40L, IL-21 and IL-4 significantly promote *in vitro* proliferation of CLL cells (6). Moreover, it is important to consider that crosstalk between BCR, CD40, and TLR signaling occurs *in vitro*, and may thus play an important role *in vivo*. Consequently, we propose that combined triggering of multiple nodes of BCR, CD40, and TLR signaling in combination with costimulatory signals by cytokines orchestrate CLL proliferation, both *in vitro* and *in vivo* (**Figure 3**).

Modeling the CLL microenvironment is an area of intense investigation, and most studies have been performed using CLL cells isolated from PB as tissue-residing CLL cells are not easily obtained. Current experimental methods relying on the investigation of PB CLL cells lack the ability to appropriately mimic the lymphoid microenvironment due to a lack of *in vitro* cultures that allow the long-term expansion of CLL cells. Crucially, this indicates that essential factors or aspects are missing in current *in vitro* models. Despite these limitations, many *in vitro* observations have helped to elucidate the role of (the combination of) individual signals in the proliferation of CLL *in vivo*, including the development of new therapies to target CLL proliferation.

New innovative *in vitro* CLL models continue to be developed and most promising could be 3D cultures that may overcome some of the current limitations of *in vitro* studies. Current 2D culture systems do not reflect the true 3D microenvironment present in human tissues, where various types of cell-cell interactions and interactions with the extracellular matrix occur, which may be fundamental to study CLL proliferation (135). Although the use of 3D models is new in the field of CLL, a recent study reported a significant increase in proliferative response, proliferation rates and number of cell generations compared to 2D cultures, irrespective of the biological characteristics of CLL cells (6). Therefore, 3D models may contribute pathophysiological relevance to *in vitro* culture systems of CLL and will be valuable for future studies. Similar to 2D CLL culture systems, many types of 3D models have been developed for solid tumors, including the use of scaffolds, gels, spheroid cultures and fluidic systems (136). However, unlike solid tumors, secondary lymphoid tissues do not derive from a single stem cell progenitor and thus the advantages and limitations of each of these systems have to be evaluated in terms of accurate mimicking of the CLL microenvironment. We may safely predict that in the near future a variety of 3D CLL systems will be reported. The ultimate goal is to implement a standardized system for *in vitro* proliferation, that will allow novel drug testing, as well as meaningful study of various CLL clonotypes.

## Author Contributions

MH wrote the review and prepared figures. EE conceptualized and wrote the review. AK edited the review and contributed text. All authors contributed to the article and approved the submitted version.

## Funding

MH was funded by the Blaauboer Fund *via* the AMC Foundation.

## Conflict of Interest

The authors declare that the research was conducted in the absence of any commercial or financial relationships that could be construed as a potential conflict of interest.
